# Small Bone Transplants in Bridging

**Published:** 1918-10

**Authors:** 


***The Use of Small Bone Transplants in Bridging a Bone Defect.*** By Dr. Frederic W. Bancroft, New York. *Annal of Surgery*, April 1918.

In certain fractures where there is malunion with loss of substance, and where the bone is too small to allow a transplant to be placed satisfactorily, the writer recommends that small fragments of bone (1/2 mm. in size) may be used.

He has experimented with a series of 26 dogs, of which two became infected, two jumped from elevations, and angular union resulted. The remaining 22 gave very satisfactory results. In specimens of one year duration it is difficult to find the bone transplants.

He feels that this procedure offers an adequate method of bridging a bone defect if there is another bone present to act as a splint, and if it is possible to hold the bone in position by external support.

